# Supplementary material for: Library Preparation Based on Transposase Assisted RNA/DNA Hybrid Co-Tagmentation for Next-Generation Sequencing of Human Noroviruses
Source: Viruses. 2021 Jan 6;13(1):65. doi: 10.3390/v13010065 (PMC7825083; doi:10.3390/v13010065)
Supplement: Supplementary file 1 [file viruses-13-00065-s001.pdf]

Table S1. Primers designed in this study.

Primer	Sequence (5′-3′)	Tube
RTGI-1	TGAATATGRTGCCC	A
RTGI-2	RTTTGCGAAYA	B
RTGI-3	GCYTGRGCCATTAT	A
RTGI-4	GTTCCCACAGGCTT	B
RTGI-6	CCARCCATTRTACAT	A
RTGI-7	TAGACGCCATCATC	B
RTGI-8	TCATCATCNCCRTA	A
RTGI-9	TTCCARTCRTCRTT	B
RTGI-10	CCARAARCCCCA	A
RTGI-11	AAYTCRTCYTG	B
RTGI-12	TGRTAKCCRTCCCA	A
RTGI-13	CCRCACATCAT	B
RTGI-14	CCCATRAANGTCCA	A
RTGII-0	TGACTCCCCYC	B
RTGII-1	GTCCAGGAGTCCA	A
RTGII-3	TTCCCATGGGGG	B
RTGII-4	TTTTGTGGTTGNAC	A
RTGII-5	TTTTGRCACTGNAC	
RTGII-6	CATCTTCATTCAC	B
RTGII-7	TCATTCATRTTCAT	A
RTGII-8	CATRCCCATYTG	B
RTGII-9	TACTCYTCATCRCT	A
RTGII-10	TTCCACATRTCWGG	B
RTGII-11	AARAACCAYTTCA	A
RTGII-12	CCRTCCCACATYTC	B
